# Long-term and effective neutralization against omicron sublineages elicited by four platform COVID-19 vaccines as a booster dose

**DOI:** 10.1038/s41421-023-00518-2

**Published:** 2023-02-08

**Authors:** Yuemiao Zhang, Meng-Ting Luo, Qingqin Wu, Yun-Xia Wang, Xupu Ma, Guanghong Yan, Si-Hang Zhang, Yanli Chen, Na Wan, Liang Zhang, Dingyun You, Jia Wei, Zijie Zhang, Tai-Cheng Zhou, Precise-CoVaccine study group, Zijie Zhang, Jia Wei, Yuemiao Zhang, Yanli Chen, Chunmei Li, Wei Yang, Hanfang Bi, Ao Li, Rong Wang, Wanting Qin, Xuanjing Yu, Zumi Zhou, Xinshuai Zhao, Xinyu Jiang, Wei Su, Tianpei Shi, Mei Yang, Yating Yan, Lei Xing, Jingmei Li, Lipei Sun, Hanyi Jiao, Junze Wu, Xueyan Liu, Houze Yu, Muxian Dai, Fengwei Liu, Muhua Feng, Jun Hu, Ying Wu, Guo-Dong Wang, Zhenwang Fu, Gangxu Xu, Yajing Wang, Lihong Zhang

**Affiliations:** 1 Central Laboratory, Liver Disease Research Center and Department of Infectious Disease, The Affiliated Hospital of Yunnan University, Kunming, Yunnan China; 2 Renal Division, Department of Medicine, Peking University First Hospital, Renal Pathology Center, Institute of Nephrology, Peking University, Key Laboratory of Renal Disease, Ministry of Health of China, Key Laboratory of CKD Prevention and Treatment, Ministry of Education of China; Research Units of Diagnosis and Treatment of Immune-Mediated Kidney Diseases, Chinese Academy of Medical Sciences, Beijing, China; 3 State Key Laboratory of Genetic Resources and Evolution, and Yunnan Laboratory of Molecular Biology of Domestic Animals, Kunming Institute of Zoology, Chinese Academy of Sciences, Kunming and College of Life Science, University of Chinese Academy of Sciences, Beijing, China; 4 State Key Laboratory for Conservation and Utilization of Bio-resource and School of Life Sciences, Yunnan University, Kunming, Yunnan China; 5 School of Public Health, Kunming Medical University, Kunming, Yunnan China; 6 Institute of Medical Biology, Chinese Academy of Medical Sciences & Peking Union Medical College, Kunming, Yunnan China; 7 Yunnan Key Laboratory of Stem Cell and Regenerative Medicine, Institute of Biomedical Engineering, Kunming Medical University, Kunming, Yunnan China; 8 Yunnan Key Laboratory of Stomatology, Kunming Medical University, Kunming, Yunnan China; 9 Liver Disease Research Center and Department of Infectious Disease, Affiliated Hospital of Yunnan University, Kunming, Yunnan China; 10 Research Units of Diagnosis and Treatment of Immune-Mediated Kidney Diseases, Chinese Academy of Medical Sciences, Beijing, China; 11 Central Laboratory, Affiliated Hospital of Yunnan University, Kunming, Yunnan China; 12 Preventive Medicine Department, Affiliated Hospital of Yunnan University, Kunming, Yunnan China; 13 Guangdong Provincial Key Laboratory of Tropical Disease Research, Department of Biostatistics, School of Public Health, Southern Medical University, Guangdong, China; 14 Hainan Center for Disease Control and Prevention, Hainan, China

**Keywords:** Autoimmunity, Molecular biology

Dear Editor,

As of July 2022, over 89% of Chinese population have finished COVID-19 vaccine primary immunization, mostly with two-dose inactivated vaccines. A booster dose has been deployed to combat waning immunity over time and immune escape due to evolving virus variants. However, previous studies revealed that homologous booster with CoronaVac/BBIBP-CorV or heterologous booster with ZF2001 (recombinant RBD subunit protein vaccine) in participants primed with two-dose CoronaVac or BBIBP-CorV induced limited cross-neutralization against the more recently prevalent omicron sublineages BA.4/BA.5^1–3^. Although heterologous booster with mRNA vaccine (BNT162b2) has been shown to boost significantly higher neutralization against BA.2 than homologous booster, neutralization against the latest omicron sublineages is lacking^4^. Till now, there is no studies comparing the boosting effects against the latest omicron sublineages by multi-platform COVID-19 vaccines comprehensively. More importantly, the long-term durability against omicron sublineages remains to be explored. Here, we report firstly the vaccination-induced cross-neutralization data against omicron sublineages, including BA.2.75 and BF.7, in head-to-head comparison of COVID-19 vaccines from four platforms within a 3-month follow-up period.

Previously, we performed an randomized controlled trial (RCT) (ChiCTR.org.cn Identifier: ChiCTR2200057758) to evaluate immunogenicity in head-to-head design of four COVID-19 vaccines in China representing four major platforms^5^, including RQ3013^6^, ChAdTS-S^7,8^, ZR202-CoV^9^, and CoronaVac. RQ3013 is a pseudouridine-modified mRNA vaccine encoding a near full-length Spike protein of alpha strain (B.1.1.7) with additional mutations from beta variant (B.1.351). ZR202-CoV is a recombinant protein vaccine based on a prefusionstabilized Spike ectodomain trimer of wild-type SARS-CoV-2 with two mutation sites, including a “GGSG” substitution at the furin cleavage site (residues 682–685) and proline substitutions at residues 986 and 987. ChAd-TS-S is a chimpanzee adenovirus serotype 68 (AdC68) vector-based vaccine encoding the full-length Spike protein of wild-type SARS-CoV-2. CoronaVac is an inactivated vaccine of the whole wild-type SARS-CoV-2 (CN02 strain) (see Supplementary Methods). In this RCT, a total of 234 participants aged 18–59 years, who had received a prime two-dose CoronaVac (3–5 weeks apart) vaccination 100–270 days before, were enrolled and randomized to receive one of the four COVID-19 vaccines or placebo as a booster dose. Their median age was 28 years with interquartile range from 24 to 34 years, and 126/234 (53.85%) were females. All their demographic characteristics and baseline immunogenicity measurements distributed comparably across five groups^5^. This trial has been performed in accordance with the Declaration of Helsinki and approved by the Committee on Human Subject Research and Ethics of The Affiliated Hospital of Yunnan University (ID: 2022026). Signed informed consent has been obtained from all participants. The results revealed varying levels of magnitude and breadth in neutralizing antibodies against live SARS-CoV-2 (wild-type, delta [B.1.617.2] and omicron [BA.1.1]) across four vaccines, with the mRNA vaccine RQ3013 demonstrating the highest levels of neutralizing antibodies^5^. In this work, we further tracked the variants of concerns (VOCs) of SARS-CoV-2 and tested neutralizing antibodies comprehensively against 8 omicron sublineages (BA.1, BA.1.1, BA.3, BA.2.13, BA.2.12.1, BA.2.75, BA.4/BA.5, and BF.7) measured by the pseudovirus test^1,10^ (see Supplementary Methods) (Fig. 1a).

**Fig. 1:**
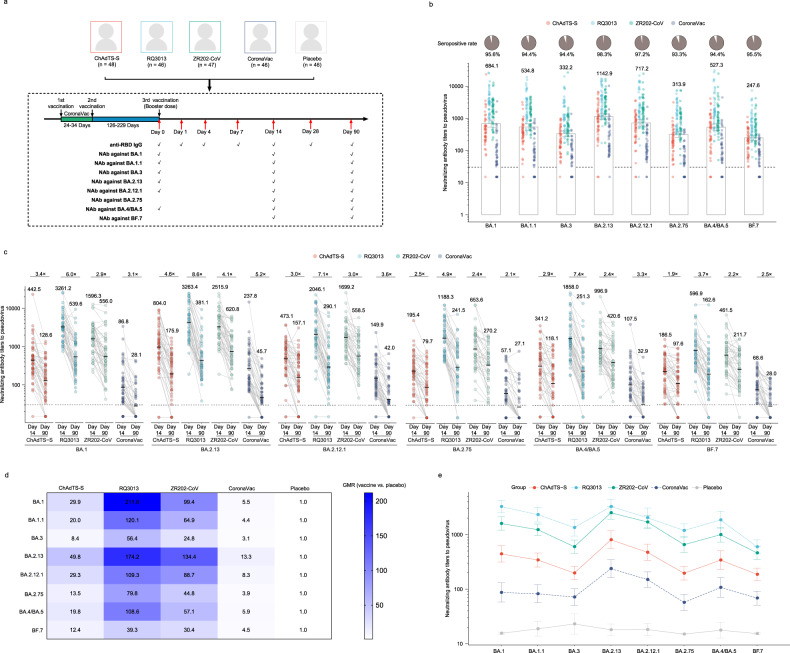
Magnitude, breadth, and durability of omicron sublineage cross-neutralization elicited by four COVID-19 vaccines as a booster dose. **a** This figure illustrates the study design and the key immunogenicity data for this randomized controlled trial. One participant receiving RQ3013 failed bleeding at day 14 after the booster dose vaccination, resulting in 233 participants in this study. **b** Neutralizing antibodies against omicron sublineages (BA.1, BA.1.1, BA.3, BA.2.13, BA.2.12.1, BA.2.75, BA.4/BA.5, and BF.7) were measured using pseudovirus test. Data are presented as the geometric mean titers (GMTs) with 95% confidence intervals (CI). The titers for individual participants are indicated with datapoints. Seroconversion rates by booster schedules are also presented. **c** Dynamic changes of neutralizing antibodies against omicron sublineages from day 14 to day 90. **d** Geometric mean ratios (GMRs) of neutralizing antibodies against omicron sublineages at day 14 after the third dose vaccination across booster regimens. GMRs were calculated using linear regression model by adjusting for the baseline neutralizing antibody levels at day 0 before the booster dose vaccination, the first and second dose interval, and the second and third dose interval by comparing to placebo. **e** GMTs of neutralizing antibodies against omicron sublineages across booster regimens are presented as the GMTs with 95% CI.

Firstly, we analyzed time-series anti-RBD specific IgG data (see Supplementary methods) before and after the booster dose to explore the dynamics of antibody responses to the booster dose (Fig. 1a). The baseline anti-RBD-specific IgG was close to seropositive cutoff across all groups until day 4. Since then, we observed a rapid increase in anti-RBD-specific IgG at day 7, a peak at day 14, a slight waning at day 28, and a retention at day 90 (Supplementary Fig. S1). Similarly, neutralizing antibodies against the omicron sublineages were close to the seroconversion cutoff in CoronaVac-primed participants 4–7 months post-vaccination (Supplementary Table S1). At day 14, in which humoral response peaked, majority (> 93%) of booster-vaccinated sera neutralized pseudovirus harboring BA.1-, BA.1.1-, BA.3-, BA.2.13-, BA.2.12.1-, BA.2.75-, BA.4/BA.5-, BF.7-SARS-CoV-2-Spike with titers > 30 (seropositive cutoff, Fig. 1b). 3 months after vaccination, we found that neutralizing antibodies against all 8 omicron sublineages, including the more recently prevalent BA.2.75, BA.4/BA.5 and BF.7 remained mostly seropositive in heterologous boost schedules while homologous boosting with CoronaVac showed less than 50% seropositivity (Fig. 1c and Supplementary Fig. S2). Interestingly, the recombinant protein vaccine ZR202-CoV with CpG as adjuvant and the adenovirus-vectored vaccine ChAdTS-S showed much slower decline rates against omicron variants compared with the mRNA vaccine RQ3013 (2–4-fold vs 4–11-fold decrease, respectively). This is consistent with previous studies that the usage of adjuvant CpG 7909 in ZR202-CoV was reported to improve immune persistence by stimulating human B cells and plasmacytoid dendritic cells^11^. The durable humoral response of adenovirus-vectored vaccine ChAdTS-S could potentially attribute to longer half-life of immunogen-expressing adenovirus vector than mRNA, which was also observed in the Ad26.COV2.S vaccine^12^. Additionally, the broad host cell tropism of the vector AdC68 and the expression of membrane-anchored rather than soluble Spike protein by ChAdTS-S might also contribute to the relative stronger durability^8^. Notably, despite of slower waning immunity for the adenovirus-vectored vaccine, our head-to-head comparison revealed much higher absolute neutralizing titers for ZR202-CoV and RQ3013 at 3 months after vaccination. Thus, ZR202-CoV and RQ3013 demonstrated superior protective capacity over time than ChAdTS-S and CoronaVac.

For each omicron sublineage, we observed a wide range of boosting effects across vaccine types by a third dose (Fig. 1d). Although the mRNA vaccine RQ3013 exhibited the highest neutralization against all 8 omicron sublineages, the superiorities varied by sublineages (Supplementary Table S2). Taking the currently prevalent variant in China BF.7 as an example, the geometric mean titers (GMTs) of neutralizing antibodies were 596.9 (95% confidence interval [CI] 443.8–802.9), 461.5 (95% CI 349.0–610.4), 186.5 (95% CI 143.6–242.2), 68.6 (95% CI 51.0–92.3) and 15.3 (95% CI 14.7–16.0) for RQ3013, ZR202-CoV, ChAdTS-S, CoronaVac and placebo, respectively. We observed similar patterns of immune escape by different omicron sublineages with significantly different neutralization capacity across boosting regimens (Fig. 1e and Supplementary Table S3). Among the 8 omicron sublineages we tested, BA.2.75 and BF.7 exhibited the most substantial immune evasion, with GMRs of 0.2–0.4 as compared to BA.1, supporting their widespread prevalence and the necessity for a booster dose with a vaccine of higher efficacy.

In this study, we mainly focused on the broadness of neutralizing antibody elicited by vaccine and several limitations should be mentioned. First, different assay systems exhibit different levels of neutralizing antibodies, and antibody titers measured by different assays are not directly comparable. However, the neutralizing antibody titers measured by our pseudovirus neutralization assay correlated well with those measured by live virus assay (Supplementary Fig. S3) and were validated using omicron variant-specific monoclonal antibody as positive controls (Supplementary Table S4). The seropositive cut-off value does not necessarily mean protection against SARS-CoV-2 infection. Second, we note that our pseudovirus neutralization test was limited by its sensitivity to compare immune escape of different variants of strain at low neutralizing level. Third, neutralizing antibodies are not the only arm of immunity against SARS-CoV-2. Cellular response, which exhibited less immune evasion across omicron sublineages^5^, was also essential for vaccine efficacy, especially for preventing disease severity^13^.

To summarize, this trial demonstrated the potential of four platform vaccines (ChAdTS-S, RQ3013, ZR202-CoV, and CoronaVac) to boost immunity against omicron sublineages following an initial course of CoronaVac/CoronaVac. Specifically, a booster dose with the mRNA RQ3013 elicited the strongest immune responses (18.3-fold higher BA.4/BA.5- and 8.8-fold higher BF.7-neutralization than 3×CoronaVac at day 14), while the recombinant protein vaccine ZR202-CoV with CpG as adjuvant optimized the durability (13.0-fold higher BA.4/BA.5- and 7.6-fold higher BF.7-neutralization than 3×CoronaVac 3 months post-vaccination) (Supplementary Table S5). In comparison, Cao et al. showed that booster dose with ZF2001 or 3×CoronaVac plus BA.1 infection elicited 1.4-fold neutralization than 3×CoronaVac against BA.4/BA.5^1^. Thus, our data suggest that RQ3013 and ZR202-CoV could likely provide more effective neutralization against omicron variants over at least 3 months compared to current alternative vaccines in China. This immunogenicity and durability data of multiple platform vaccines provide insights into the additional booster dose vaccination strategy for optimal breadth and duration of protection against the current and future sub-variants.

## Supplementary information

Supplementary Figures and Tables

## Data Availability

De-identified data are freely available from the corresponding author upon request.

## Code availability

Codes associated with data analysis are freely available from the corresponding author upon request.
