# Supplementary material for: Long-term and effective neutralization against omicron sublineages elicited by four platform COVID-19 vaccines as a booster dose
Source: Cell Discov. 2023 Feb 8;9:17. doi: 10.1038/s41421-023-00518-2 (PMC9907876; doi:10.1038/s41421-023-00518-2)
Supplement: Supplementary file 1 — Supplementary Figures and Tables [file 41421_2023_518_MOESM1_ESM.pdf]

Supplementary information

Long-term and effective neutralization against omicron sublineages elicited by four platform COVID-19 vaccines as a booster dose

Materials and Methods

1. Studying vaccines

Four COVID-19 vaccines representing four major platforms were used in this study, including the mRNA vaccine RQ3013, the adenovirus-vectored vaccine ChAdTS-S, the recombinant protein vaccine ZR202-CoV and the inactivated vaccine CoronaVac. More characteristics of these vaccines were shown in **Table 1**.

Table 1. Action mechanism of the four platform COVID-19 vaccines in this study.

COVID-19 vaccines	Antigens	Mutations	Components with adjuvant activity
RQ3013	Pseudouridine-modified mRNA encodes a near full-length Spike protein of alpha strain (B.1.1.7), which also introduces mutations from beta variant (B.1.351).	ΔH69, ΔV70, ΔY144, A570D, P681H, T761I, S982A and D118H from the alpha variant (B.1.1.7), and K417N, E484K, N501Y, D614G and A701V from the beta variant (B.1.351), seven of which (ΔH69, ΔV70, ΔY144, K417N, N501Y, D614G and P681H) were shared with the omicron variant (B.1.1.529).	Lipid nanoparticle (LNP)
ZR202-CoV	Prefusionstabilized Spike ectodomain trimer of wild-type SARS-CoV-2 (GenBank: MN908947) with two mutation sites.	A "GGSG" substitution at the furin cleavage site (residues 682-685) and proline substitutions at residues 986 and 987.	Aluminium hydroxide and CpG 7909
ChAd-TS-S	The full-length Spike protein of wild-type SARS-CoV-2 (GenBank: MN908947.3).	NA	NA
CoronaVac	Whole, inactivated wild-type SARS-CoV-2 (CN02 strain).	NA	Aluminium hydroxide

2. Measurement of anti-SARS-CoV-2 RBD-specific IgG

Given the widespread accessibility of anti-SARS-CoV-2 spike RBD serological assays in the clinical setting, we evaluated titers of SARS-CoV-2 RBD specific IgG at intensive time points to explore the kinetics of humoral responses.

Specifically, we used magnetic particle chemiluminescence immunoassay (MCLIA) (Bioscience Co., China)[1,2] to measure anti-SARS-CoV-2 RBD-specific IgG tires. Antibody tires were presented as the measured chemiluminescence values tracing to the international standard (binding antibody unit [BAU]/mL, NIBSC code: 20/136). The cutoff value of this test was defined by the receiver operating characteristic curves. We tested the antibody tire serum sample except for samples that exhibit neutralizing level higher than 250 BAU/mL (the upper bound of linearity for antibody detection). For these samples, we tested series-diluted samples until the measured values fall within the detection range. Seroconversion cutoff was defined as 5 BAU/mL for IgG according to the kit manufacturer.

3. Pseudovirus neutralization assay

The pseudovirus neutralization assay was preformed according to previous studies [3,4]. Briefly, the spike protein of omicron sublineages (BA.1, BA.1.1, BA.3, BA.2.13, BA.2.12.1, BA.2.75, BA.4/BA.5 and BF.7) were constructed into pcDNA3.1 vector, respectively (**Table 2**). 20 μg of each construct was transfected into HEK-293T cells in 10 cm dish with Lipo293™ transfection reagent (Beyotime, C0521). VSV*ΔG-Rluc pseudovirus which was gifted from professor Yong-Tang Zheng's research group was added 24 h after transfection with 7×10^4 TCID50. After 8 h of infection, the VSV-ΔG-Rluc residues were removed and washed three times by PBS which contain 1% fetal bovine serum. Then we added 10 ml of fresh complete DMEM (BasalMedia, L110KJ) to the dish. After the incubation in a 5% CO_2 incubator at 37°C for 24-30 h, the supernatants containing SARS-CoV-2 pseudovirus was harvested, filtered through a 0.45μm filter (Millipore, SLHV033RB), and then aliquoted and stored them at -80°C for further use. The VSV*ΔG-Rluc pseudovirus packaged by pCMV-VSV-G plasmid with the same method.

Titration of the SARS-CoV-2 pseudovirus detection was performed in 293T cells overexpressing human angiotensin-converting enzyme 2 (293T-hACE2 cells). For the neutralization assay, the serum samples were first heat-inactivated at 56°C for 30 min. Then, starting from 1:10 for baseline samples or 1:30 for other samples, the samples were serially diluted three-fold in DMEM (BasalMedia, L110KJ) on a 96-well white plate (Beyotime, FCP968). The SARS-CoV-2 Spike antibody (SinoBiological, 40592-MM117) and SARS-CoV-2 omicron (BA.4/BA.5/BA.2.75) Spike RBD specific antibody were used as positive controls, starting from 20 μg/ml with the same method. The SARS-CoV-2 pseudoviruses were added at 1.3×10^4 TCID50 and incubated with the diluted sera at 5% CO_2 and 37°C for 1 h. After the incubation, we added 100μl of 293T-hACE2 cells with 2

× 10^5 cells/ml into virus-serum mixtures. After 20-28 hours of culture, 150μl supernatants were discarded and added 100μl Renilla-Lumi™reagent (Beyotime, RG066M), and the luminescence value was detected using a microplate spectrophotometer (Thermo Scientific Varioskan LUX, USA). The amount of pseudovirus entering the target cells was calculated by detecting the expression of luciferase to obtain the neutralizing antibody content of the sample. The infection inhibition rates of each dilution of the sample were calculated according to the RLU values as follows: inhibition rate=[1 − (average RLU of sample − average RLU of negative control) / (average RLU of positive control − average RLU of negative control)] × 100%. The EC_{50} of each sample is calculated by the Reed-Muench method.

Table 2. Spike protein mutations of omicron sublineages.

**Omicron sublineages**	**Mutations**
BA.1	A67V, H69del, V70del, T95I, G142D, V143del, Y144del, Y145del, N211del, L212I, ins214EPE, G339D, S371L, S373P, S375F, K417N, N440K, G446S, S477N, T478K, E484A, Q493R, G496S, Q498R, N501Y, Y505H, T547K, D614G, H655Y, N679K, P681H, N764K, D796Y, N856K, Q954H, N969K, L981F
BA.1.1	BA.1+ R346K
BA.2.12.1	BA.2+L452Q+S704L
BA.2.13	BA.2+L452M
BA.3	A67V, del69-70, T95I, G142D, V143del, Y144del, Y145del, N211del, L212I, G339D, S371F, S373P, S375F, D405N, K417N, N440K, G446S, S477N, T478K, E484A, Q493R, Q498R, N501Y, Y505H, D614G, H655Y, N679K, P681H, N764K, D796Y, Q954H, N969K
BA.2.75	T19I, del24-26, A27S, G142D, K147E, W152R, F157L, I210V, V213G, G257S, G339H, S371F, S373P, S375F, T376A, D405N, R408S, K417N, N440K, G446S, N460K, S477N, T478K, E484A, R493Q, Q498R, N501Y, Y505H, D614G, H655Y, N679K, P681H, N764K, D796Y, Q954H, N969K
BA.4/5	T19I, L24S, del25-27, del69-70, G142D, V213G, G339D, S371F, S373P, S375F, T376A, D405N, R408S, K417N, N440K, G446S, L452R, S477N, T478K, E484A, F486V, Q498R, N501Y, Y505H, D614G, H655Y, N679K, P681H, N764K, D796Y, Q954H, N969K
BF.7	T19I, L24S, del25-27, del69-70, G142D, V213G, G339D, R346T, S371F, S373P, S375F, T376A, D405N, R408S, K417N, N440K, L452R, S477N, T478K, E484A, F486V, Q498R, N501Y, Y505H, D614G, H655Y, N679K, P681H, N764K, D796Y, Q954H, N969K

References

1 Long, Q. X. *et al.* Antibody responses to SARS-CoV-2 in patients with COVID-19. *Nat Med* **26**, 845-848, doi:10.1038/s41591-020-0897-1 (2020).

2 Xu, X. *et al.* Seroprevalence of immunoglobulin M and G antibodies against SARS-CoV-2 in China. *Nat Med* **26**, 1193-1195, doi:10.1038/s41591-020-0949-6 (2020).

3 Nie, J. *et al.* Quantification of SARS-CoV-2 neutralizing antibody by a pseudotyped virus-based assay. *Nat Protoc* **15**, 3699-3715, doi:10.1038/s41596-020-0394-5 (2020).

4 Cao, Y. *et al.* BA.2.12.1, BA.4 and BA.5 escape antibodies elicited by Omicron infection. *Nature* **608**, 593-602, doi:10.1038/s41586-022-04980-y (2022).

Supplementary Figures and Tables

Fig. S1 Kinetics of anti-RBD IgG after the third dose vaccination by booster schedules.

Fig S2. Cross-neutralization durability of BA.1.1 and BA.3 elicited by four COVID-19 vaccines as a booster dose.

Fig. S3 Correlation analyses of neutralizing antibodies against omicron sublineages measured by two independent methods.

Table S1. Neutralizing antibody titers against omicron sublineages of pseudovirus at baseline day 0 before the booster dose vaccination.

Table S2. Geometric mean titer of neutralizing antibodies against omicron sublineages at day 14 after the third dose vaccination and geometric mean ratio comparing heterologous and homologous booster regimens.

Table S3. Omicron sublineage cross-neutralization as shown by geometric mean ratio comparing neutralization against omicron BA.1.

Table S4. Positive control experiments on monoclonal antibodies against omicron sublineages.

Table S5. Geometric mean titer of neutralizing antibodies against omicron sublineages at day 90 after the third dose vaccination and geometric mean ratio comparing heterologous and homologous booster regimens.

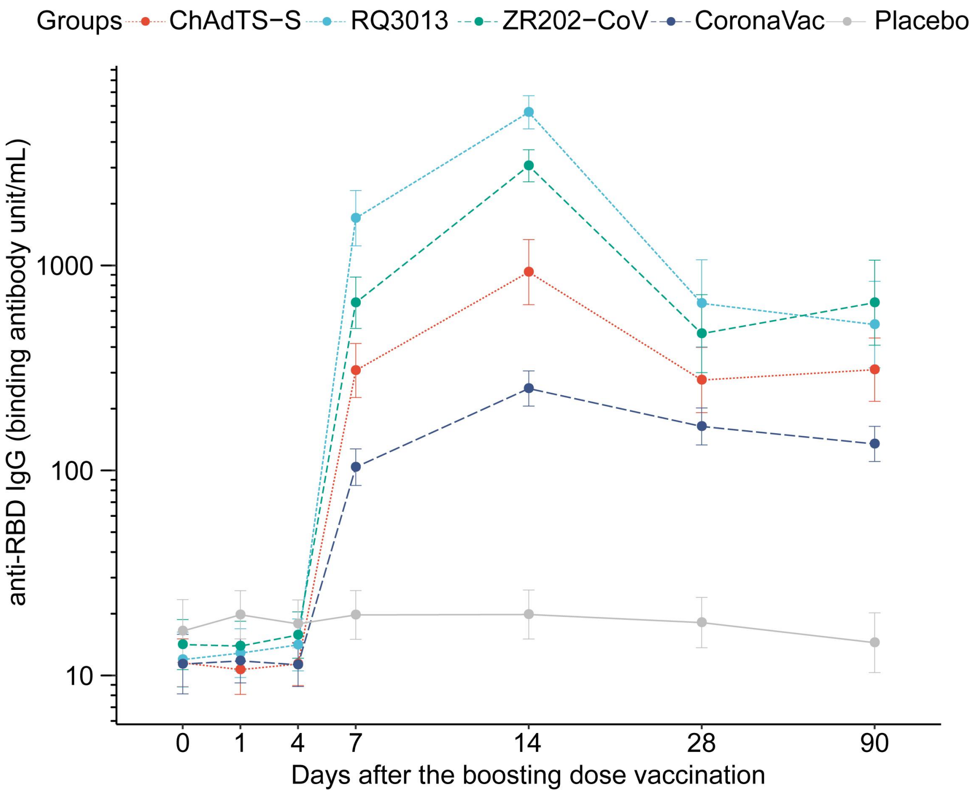

Fig. S1 Kinetics of anti-RBD IgG after the third dose vaccination by booster schedules. The anti-RBD specific IgG titers were measured by magnetic particle chemiluminescence immunoassay (MCLIA) (Bioscience Co., China).

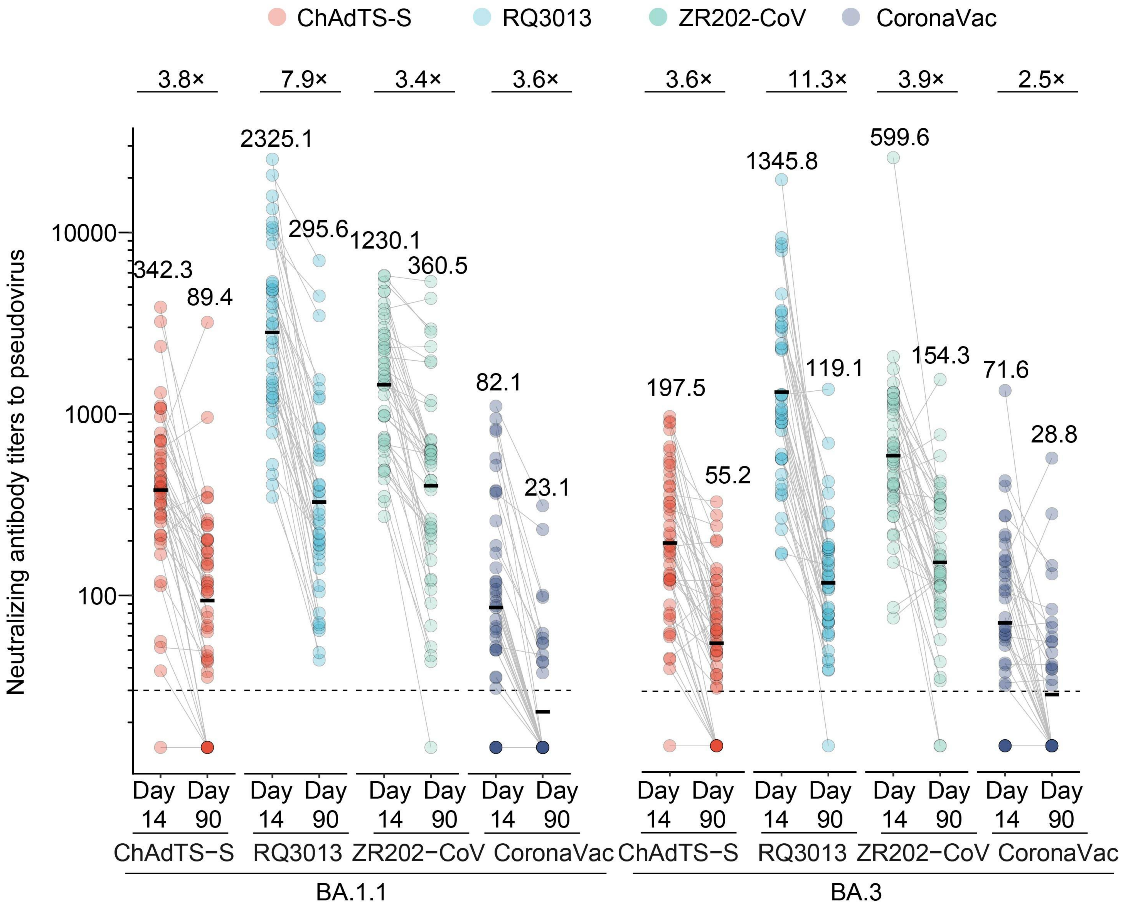

Fig S2. Cross-neutralization durability of BA.1.1 and BA.3 elicited by four COVID-19 vaccines as a booster dose. The geometric mean titers of neutralizing antibodies against BA.1.1 and BA.3 from day 14 to day 90 were presented.

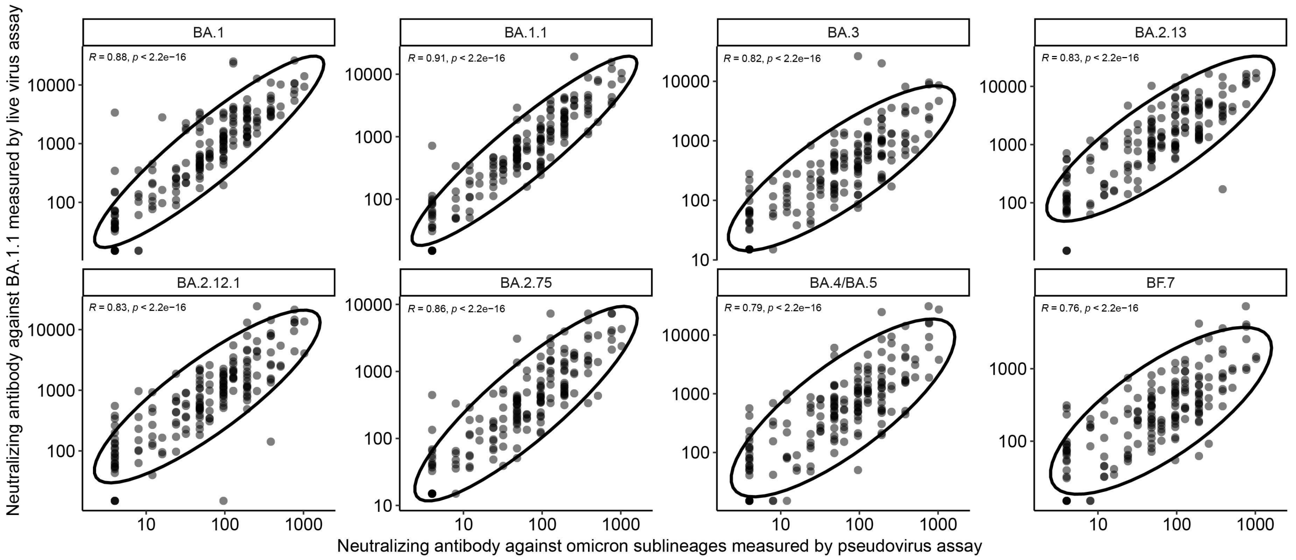

Fig. S3 Correlation analyses of neutralizing antibodies against omicron sublineages measured by two independent methods. The X-axis indicates neutralizing antibodies against BA.1, BA.1.1, BA.3, BA.2.13, BA.2.12.1, BA.2.75, BA.4/BA.5 and BF.7 sublineages of SARS-CoV-2 measured by pseudovirus assay. The Y-axis indicates neutralizing antibodies against BA.1.1 sublineage of live SARS-CoV-2 determined by cytopathic effect (CPE)-based microneutralisation assay.

Table S1. Neutralizing antibody titers against omicron sublineages of pseudovirus at baseline day 0 before the booster dose vaccination.

Omicron sublineages	ChAdTS-S (n=48)	RQ3013 (n=46)	ZR202-CoV (n=47)	CoronaVac (n=46)	Placebo （n=46）	p
BA.1	15.0(15.0,15.0)	15.0(15.0,15.0)	15.0(15.0,15.0)	15.0(15.0,15.0)	15.0(15.0,15.0)	0.4402
BA.1.1	15.0(15.0,15.0)	15.0(15.0,15.0)	15.0(15.0,15.0)	15.0(15.0,15.0)	15.0(15.0,15.0)	0.4402
BA.3	15.0(15.0,15.0)	15.0(15.0,15.0)	15.0(15.0,15.0)	15.0(15.0,15.0)	15.0(15.0,15.0)	0.7259
BA.2.13	15.0(15.0,15.0)	15.0(15.0,15.0)	15.0(15.0,15.0)	15.0(15.0,15.0)	15.0(15.0,15.0)	0.4402
BA.2.12.1	15.0(15.0,15.0)	15.0(15.0,15.0)	15.0(15.0,15.0)	15.0(15.0,15.0)	15.0(15.0,15.0)	0.4402
BA.4/BA.5	15.0(15.0,15.0)	15.0(15.0,15.0)	15.0(15.0,15.0)	15.0(15.0,15.0)	15.0(15.0,15.0)	-

Note: Neutralizing antibody titers against omicron sublineages, including BA.1, BA.1.1, BA.3, BA.2.13, BA.2.12.1 and BA.4/BA.5, were measured using pseudovirus test. Seropositivity was defined as titer ≥ 1:30.

Table S2. Geometric mean titer of neutralizing antibodies against omicron sublineages at day 14 after the third dose vaccination and geometric mean ratio comparing heterologous and homologous booster regimens.

	ChAdTS-S (n=48)	RQ3013 (n=46)	ZR202-CoV (n=47)	CoronaVac (n=46)	Placebo (n=46)
**Neutralizing antibody against the omicron sublineage BA.l**					
GMT	442.5(312.8-626.0)	3261.2(2525.8-4210.7)	1596.3(1201.1-2121.7)	86.8(57.8-130.4)	15.5(14.8-16.3)
GMR1	29.9(19.8-45.0)	211.8(141.5-317.1)	99.4(66.4-148.7)	5.5(3.7-8.2)	Ref
GMR2	5.4(3.6-8.2)	38.6(25.7-57.8)	18.1(12.1-27.1)	Ref	-
p1	<0.0001	<0.0001	<0.0001	<0.0001	Ref
p2	<0.0001	<0.0001	<0.0001	Ref	-
**Neutralizing antibody against the omicron sublineage BA.l.1**					
GMT	342.3(254.7-460.1)	2325.1(1692.1-3194.8)	1230.1(969.3-1561)	82.1(56.0-120.6)	18.7(13.7-25.6)
GMR1	20.0(12.9-30.9)	120.1(77.6-185.7)	64.9(42.2-99.9)	4.4(2.8-6.7)	Ref
GMR2	4.6(2.9-7.1)	27.4(17.7-42.5)	14.8(9.6-22.8)	Ref	-
p1	<0.0001	<0.0001	<0.0001	<0.0001	Ref
p2	<0.0001	<0.0001	<0.0001	Ref	-
**Neutralizing antibody against the omicron sublineage BA.3**					
GMT	197.5(149.3-261.4)	1345.8(945.2-1916.1)	599.6(453.3-793.1)	71.6(50.2-102.1)	23.0(15-35.4)
GMR1	8.4(5.2-13.4)	56.4(35.3-90.0)	24.8(15.6-39.5)	3.1(1.9-5.0)	Ref
GMR2	2.7(1.7-4.3)	18.0(11.2-29.0)	7.9(5.0-12.7)	Ref	-
p1	<0.0001	<0.0001	<0.0001	<0.0001	Ref
p2	<0.0001	<0.0001	<0.0001	Ref	-
**Neutralizing antibody against the omicron sublineage BA2.13**					
GMT	804.0(551.1-1172.9)	3263.4(2386.1-4463.2)	2515.9(1918.3-3299.6)	237.8(163.1-346.8)	18.0(14.2-22.9)
GMR1	49.8(32.0-77.5)	174.2(112.4-270.1)	134.4(86.9-207.8)	13.3(8.6-20.7)	Ref
GMR2	3.7(2.4-5.8)	13.1(8.4-20.4)	10.1(6.5-15.7)	Ref	-
p1	<0.0001	<0.0001	<0.0001	<0.0001	Ref
p2	<0.0001	<0.0001	<0.0001	Ref	-
**Neutralizing antibody against the omicron sublineage BA2.12.1**					
GMT	473.1(335.3-667.5)	2046.1(1356.0-3087.5)	1699.2(1291.6-2235.6)	149.9(108.3-207.6)	18.2(14.0-23.5)
GMR1	29.3(18.5-46.3)	109.3(69.6-171.8)	88.7(56.5-139.3)	8.3(5.3-13.1)	Ref
GMR2	3.5(2.2-5.6)	13.1(8.3-20.6)	10.6(6.8-16.7)	Ref	-
p1	<0.0001	<0.0001	<0.0001	<0.0001	Ref
p2	<0.0001	<0.0001	<0.0001	Ref	-
**Neutralizing antibody against the omicron sublineage BA.2.75**					
GMT	195.4(146.3-260.8)	1188.3(894.7-1578.4)	653.6(469.1-910.7)	57.1(40.5-80.3)	15.0 (15.0-15.0)
GMR1	13.5(9.2-19.7)	79.8(54.5-116.9)	44.8(30.5-65.8)	3.9(2.6-5.7)	Ref
GMR2	3.5(2.4-5.1)	20.7(14.0-30.6)	11.6(7.8-17.2)	Ref	-
p1	<0.0001	<0.0001	<0.0001	<0.0001	Ref
p2	<0.0001	<0.0001	<0.0001	Ref	-
**Neutralizing antibody against the omicron sublineage BA.4/BA.5**					
GMT	341.2(230.6-504.8)	1858.0(1283.6-2689.3)	996.9(723.5-1373.7)	107.5(71.2-162.5)	17.7(12.7-24.7)
GMR1	19.8(12.0-32.9)	108.6(65.4-180.4)	57.1(34.4-94.8)	5.9(3.5-10.0)	Ref
GMR2	3.3(2.0-5.6)	18.3(11.0-30.6)	9.6(5.8-16.1)	Ref	-
p1	<0.0001	<0.0001	<0.0001	<0.0001	Ref
p2	<0.0001	<0.0001	<0.0001	Ref	-
**Neutralizing antibody against the omicron sublineage BF.7**					

GMT	186.5(143.6-242.2)	596.9(443.8-802.9)	461.5(349.0-610.4)	68.6(51.0-92.3)	15.3(14.7-16.0)
GMR1	12.4(8.7-17.6)	39.3(27.6-55.8)	30.4(21.4-43.4)	4.5(3.1-6.4)	Ref
GMR2	2.8(1.9-3.9)	8.8(6.1-12.6)	6.8(4.8-9.7)	Ref	-
p1	<0.0001	<0.0001	<0.0001	<0.0001	Ref
p2	<0.0001	<0.0001	<0.0001	Ref	-

Note: Geometric mean ratio (GMR) was calculated using linear regression model by adjusting for the baseline neutralizing antibody levels at day 0 before the booster dose vaccination, the first and second dose interval, and the second and third dose interval. GMR1 and p1 were calculated by comparing to placebo group, while GMR2 and p2 were calculated by comparing to the CoronaVac group.

Table S3. Omicron sublineage cross-neutralization as shown by geometric mean ratio comparing neutralization against omicron BA.1.

SARS-CoV-2 Variants	ChAdTS-S(N=48)			RQ3013(N=46)			ZR202-CoV(N=47)			CoronaVac(N=46)			Placebo(N=46)		
	GMT (95% CI)	GMR (95% CI)	p	GMT (95% CI)	GMR (95% CI)	p	GMT (95% CI)	GMR (95% CI)	p	GMT (95% CI)	GMR (95% CI)	p	GMT (95% CI)	GMR (95% CI)	p
**BA.1**	442.5 (312.8-626.0)	Ref	Ref	3261.2 (2525.8-4210.7)	Ref		1596.3 (1201.1-2121.7)	Ref	Ref	86.8 (57.8-130.4)	Ref	Ref	15.5 (14.8-16.3)	Ref	Ref
**BA.1.1**	342.3 (254.7-460.1)	0.8 (0.5-1.2)	0.2168	2325.1 (1692.1-3194.8)	0.7 (0.5-1.1)	0.1001	1230.1 (969.3-1561)	0.8 (0.5-1.2)	0.2198	82.1 (56.0-120.6)	0.9 (0.6-1.4)	0.7961	18.7 (13.7-25.6)	1.2 (0.8-1.8)	0.3783
**BA.3**	197.5 (149.3-261.4)	0.4 (0.3-0.7)	0.0002	1345.8 (945.2-1916.1)	0.4 (0.3-0.6)	<0.0001	599.6 (453.3-793.1)	0.4 (0.2-0.6)	<0.0001	71.6 (50.2-102.1)	0.8 (0.5-1.3)	0.4204	23.0 (15-35.4)	1.5 (1-2.3)	0.0719
**BA.2.13**	804.0 (551.1-1172.9)	1.9 (1.2-2.8)	0.0046	3263.4 (2386.1-4463.2)	1.0 (0.7-1.5)	0.9812	2515.9 (1918.3-3299.6)	1.5 (1-2.3)	0.0401	237.8 (163.1-346.8)	2.7 (1.8-4.2)	<0.000 1	18.0 (14.2-22.9)	1.2 (0.8-1.8)	0.4851
**BA.2.12.1**	473.1 (335.3-667.5)	1.1 (0.7-1.7)	0.6735	2046.1 (1356-3087.5)	0.6 (0.4-1)	0.0286	1699.2 (1291.6-2235.6)	1.0 (0.7-1.6)	0.8939	149.9 (108.3-207.6)	1.7 (1.1-2.7)	0.0129	18.2 (14-23.5)	1.2 (0.8-1.8)	0.471
**BA.2.75**	195.4 (146.3-260.8)	0.4 (0.3-0.6)	<0.0001	1188.3 (894.7-1578.4)	0.4 (0.2-0.5)	<0.0001	653.6 (469.1-910.7)	0.4 (0.3-0.6)	<0.0001	57.1 (40.5-80.3)	0.7 (0.4-1.0)	0.0363	15.0 (15.0-15.0)	1.0 (0.7-1.4)	0.8678
**BA.4/BA.5**	341.2 (230.6-504.8)	0.8 (0.5-1.2)	0.2434	1858.0 (1283.6-2689.3)	0.6 (0.4-0.9)	0.0157	996.9 (723.5-1373.7)	0.6 (0.4-0.9)	0.0218	107.5 (71.2-162.5)	1.2 (0.8-2.0)	0.3678	17.7 (12.7-24.7)	1.1 (0.7-1.8)	0.5717
**BF.7**	186.5 (143.6-242.2)	0.4 (0.3-0.6)	<0.0001	596.9 (443.8-802.9)	0.2 (0.1-0.3)	<0.0001	461.5 (349.0-610.4)	0.3 (0.2-0.4)	<0.0001	68.6 (51.0-92.3)	0.8 (0.5-1.2)	0.2277	15.3 (14.7-16.0)	1.0 (0.7-1.5)	0.9507

Note: Geometric mean ratio (GMR) was calculated using linear regression model by adjusting for the baseline neutralizing antibody levels at day 0 before the booster dose vaccination, the first and second dose interval, and the second and third dose interval by comparing to the primary omicron sub-variant BA.1.

Table S4. Positive control experiments on monoclonal antibodies against omicron sublineages.

Monoclonal antibody	Neutralizing antibody titers measured using pseudovirus neutralization assay			
	**BA.1**	**BA.4/BA.5**	**BA.2.75**	**BF.7**
SARS-CoV-2 Spike Antibody (SinoBiological, 40592-MM117)	788.4162322	38.60738651	<30	<30
SARS-CoV-2 Omicron (BA.4/BA.5/BA.2.75) Spike RBD Specific Antibody (SinoBiological, 40589-D003)	<30	>7290	210.2099961	>7290

Note: the monoclonal SARS-CoV-2 Spike antibodies were tested from 20 µg/ml.

Table S5. Geometric mean titer of neutralizing antibodies against omicron sublineages at day 90 after the third dose vaccination and geometric mean ratio comparing heterologous and homologous booster regimens.

	ChAdTS-S (n=48)	RQ3013 (n=46)	ZR202-CoV (n=47)	CoronaVac (n=46)	Placebo (n=46)
**Neutralizing antibody against the omicron sublineage BA.l**					
GMT	128.6(89.3-185.3)	539.6(383.2-759.8)	556.0(378.9-815.9)	28.1(20.7-38.0)	15.0(15.0-15.0)
GMR1	8.9(5.8-13.8)	36.0(23.2-56.1)	38.1(24.6-59.1)	1.9(1.2-2.9)	Ref
GMR2	4.8(3.1-7.4)	19.3(12.4-30.0)	20.4(13.1-31.6)	Ref	-
p1	<0.0001	<0.0001	<0.0001	0.0062	Ref
p2	<0.0001	<0.0001	<0.0001	Ref	-
**Neutralizing antibody against the omicron sublineage BA.l .1**					
GMT	89.4(64.1-124.6)	295.6(207.9-420.2)	360.5(251.8-516)	23.1(18.2-29.4)	15.8(14.2-17.5)
GMR1	5.9(3.9-8.8)	19.0(12.5-28.8)	23.3(15.5-35.1)	1.5(1.0-2.2)	Ref
GMR2	4.0(2.7-6.1)	13.1(8.6-19.9)	16.0(10.7-24.2)	Ref	-
p1	<0.0001	<0.0001	<0.0001	0.0778	Ref
p2	<0.0001	<0.0001	<0.0001	Ref	-
**Neutralizing antibody against the omicron sublineage BA.3**					
GMT	55.2(42.7-71.4)	119.1(92.6-153.1)	154.3(116.9-203.6)	28.8(21.5-38.6)	19.8(16.4-23.9)
GMR1	2.8(2.0-4.0)	6.1(4.2-8.7)	7.8(5.5-11.1)	1.4(1.0-2.1)	Ref
GMR2	1.9(1.4-2.8)	4.2(2.9-6.1)	5.4(3.8-7.7)	Ref	-
p1	0.0003	<0.0001	<0.0001	0.0473	Ref
p2	<0.0001	<0.0001	<0.0001	Ref	-
**Neutralizing antibody against the omicron sublineage BA2.13**					
GMT	175.9(122.4-252.7)	381.1(275.8-526.4)	620.8(455.4-846.4)	45.7(33.8-61.9)	17.8(14.8-21.4)
GMR1	10.4(6.8-15.7)	21.6(14.2-33.0)	36.1(23.8-54.8)	2.6(1.7-4.0)	Ref
GMR2	4.0(2.6-6.1)	8.4(5.5-12.8)	14.0(9.2-21.3)	Ref	-
p1	<0.0001	<0.0001	<0.0001	<0.0001	Ref
p2	<0.0001	<0.0001	<0.0001	Ref	-
**Neutralizing antibody against the omicron sublineage BA2.12.1**					
GMT	157.1(103.9-237.5)	290.1(194.9-431.7)	558.5(404.7-770.9)	42.0(30.4-58)	16.5(14.1-19.2)
GMR1	10.1(6.4-16.0)	17.6(11.1-28.0)	35.6(22.5-56.3)	2.6(1.6-4.1)	Ref
GMR2	3.9(2.5-6.2)	6.8(4.3-10.9)	13.8(8.7-21.8)	Ref	-
p1	<0.0001	<0.0001	<0.0001	0.0001	Ref
p2	<0.0001	<0.0001	<0.0001	Ref	-
**Neutralizing antibody against the omicron sublineage BA.2.75**					
GMT	79.7(55.7-114.2)	241.5(162.6-358.6)	270.2(191.5-381.1)	27.1(19.6-37.5)	15.0 (15.0-15.0)
GMR1	5.4(3.5-8.2)	16.0(10.4-24.4)	18.2(11.7-28.1)	1.8(1.2-2.8)	Ref
GMR2	2.9(1.9-4.6)	8.8(5.6-13.7)	10.0(6.3-15.8)	Ref	-
p1	<0.0001	<0.0001	<0.0001	0.0085	Ref
p2	<0.0001	<0.0001	<0.0001	Ref	-
**Neutralizing antibody against the omicron sublineage BA.4/BA.5**					
GMT	118.1(76.7-181.9)	251.3(162.5-388.6)	420.6(295.8-598.2)	32.9(24.5-44.2)	16.8(13.8-20.4)
GMR1	7.2(4.4-11.9)	15.0(9.1-24.7)	25.5(15.5-42.2)	2.0(1.2-3.3)	Ref
GMR2	3.7(2.2-6.1)	7.6(4.6-12.6)	13.0(7.8-21.5)	Ref	-
p1	<0.0001	<0.0001	<0.0001	0.0099	Ref
p2	<0.0001	<0.0001	<0.0001	Ref	-

**Neutralizing antibody against the omicron sublineage BF.7**					
GMT	97.6(69.1-137.8)	162.6(112.1-235.9)	211.7(156.5-286.3)	28.0(21.9-35.9)	15.0 (15.0-15.0)
GMR1	6.5(4.4-9.6)	10.9(7.3-16.1)	14.1(9.5-20.9)	1.9(1.3-2.8)	Ref
GMR2	3.5(2.3-5.2)	5.9(3.9-8.7)	7.6(5.1-11.3)	Ref	-
p1	<0.0001	<0.0001	<0.0001	0.0021	Ref
p2	<0.0001	<0.0001	<0.0001	Ref	-

Note: Geometric mean ratio (GMR) was calculated using linear regression model by adjusting for the baseline neutralizing antibody levels at day 0 before the booster dose vaccination, the first and second dose interval, and the second and third dose interval. GMR1 and p1 were calculated by comparing to placebo group, while GMR2 and p2 were calculated by comparing to the CoronaVac group.
